# Interments in the City and Liberties of Philadelphia, from the 5th of June, to the 12th, 1841

**Published:** 1841-06-19

**Authors:** 


### HEALTH OF THE CITY.

INTERMENTS *in the City and Liberties of Philadelphia, from the 5th of June, to the 12th,* 1841.

| Diseases. | Adults. | Children. | Diseases. | Adults. | Children. |
|---|---|---|---|---|---|
| Apoplexy, | 1 | 0 | *Brought forward,* | 29 | 40 |
| Cancrum Oris, | 0 | 2 | Measles, | 0 | 5 |
| Cancer, | 1 | 0 | Mania a potu, | 1 | 0 |
| Caries of spine, | 0 | 1 | Neglect, | 1 | 1 |
| Casualty, | 0 | 1 | Old age, | 3 | 0 |
| Consumption of | | | Palsy, | 1 | 0 |
| the lungs, | 10 | 4 | Rheumatism, | 1 | 0 |
| Convulsions, | 0 | 4 | Rupture of a | | |
| Diarrhœa, | 0 | 1 | blood vessel, | 1 | 0 |
| Dropsy, Abdomi- | | | Small pox, | 2 | 3 |
| nal, | 1 | 0 | Still-born, | 0 | 8 |
| —— head, | 0 | 9 | Suicide, | 1 | 0 |
| Disease of the | | | Summer Com- | | |
| brain, | 1 | 0 | plaint, | 0 | 2 |
| —— bowels, | 0 | 1 | Tetanus, | 1 | 0 |
| Drowned, | 0 | 1 | Ulcerated sore | | |
| Debility, | 1 | 2 | throat, | 1 | 0 |
| Excessive heat, | 1 | 0 | Unknown, | 1 | 2 |
| Fever, | 1 | 0 | Worms, | 0 | 1 |
| —— remittent, | 1 | 1 | Wounds, | 1 | 0 |
| —— bilious, | 0 | 1 | | — | — |
| —— typhoid, | 1 | 0 | Total, 108— | 44 | 64 |
| —— Scarlet, | 0 | 2 | | | |
| Hernia, | 2 | 0 | Of the above, there | | |
| Hæmorrhage | | | were under 1 year, | | 24 |
| from bowels, | 1 | 0 | From 1 to 2 | | 13 |
| Inflammation of | | | 2 to 5 | | 17 |
| the Brain, | 1 | 0 | 5 to 10 | | 8 |
| —— Bronchi, | 0 | 1 | 10 to 15 | | 1 |
| —— Lungs, | 1 | 5 | 15 to 20 | | 1 |
| —— Stomach, | 1 | 1 | 20 to 30 | | 15 |
| —— Stomach, | | | 30 to 40 | | 3 |
| and Bowels, | 1 | 0 | 40 to 50 | | 11 |
| —— Bowels, | 2 | 1 | 50 to 60 | | 4 |
| —— Breast, | 0 | 1 | 60 to 70 | | 5 |
| —— Pleura, | 0 | 1 | 70 to 80 | | 4 |
| Jaundice, | 1 | 0 | 80 to 90 | | 2 |
| Marasmus, | 0 | 2 | 90 to 100 | | 0 |
| | — | — | | | —— |
| *Carried forward,* | 29 | 40 | Total, | | 108 |

Of the above there were 2 from the almshouse, 15 people of colour, and one from the country, which are included in the total amount.

